# Sensitivity of Ablation Targets Prediction to Electrophysiological Parameter Variability in Image-Based Computational Models of Ventricular Tachycardia in Post-infarction Patients

**DOI:** 10.3389/fphys.2019.00628

**Published:** 2019-05-24

**Authors:** Dongdong Deng, Adityo Prakosa, Julie Shade, Plamen Nikolov, Natalia A. Trayanova

**Affiliations:** ^1^Department of Biomedical Engineering, Johns Hopkins University, Baltimore, MD, United States; ^2^School of Biomedical Engineering, Dalian University of Technology, Dalian, China

**Keywords:** ventricular tachycardia, patient-specific modeling, ablation, uncertainty, LGE-MRI

## Abstract

Ventricular tachycardia (VT), which could lead to sudden cardiac death, occurs frequently in patients with myocardial infarction. Computational modeling has emerged as a powerful platform for the non-invasive investigation of lethal heart rhythm disorders in post-infarction patients and for guiding patient VT ablation. However, it remains unclear how VT dynamics and predicted ablation targets are influenced by inter-patient variability in action potential duration (APD) and conduction velocity (CV). The goal of this study was to systematically assess the effect of changes in the electrophysiological parameters on the induced VTs and predicted ablation targets in personalized models of post-infarction hearts. Simulations were conducted in 5 patient-specific left ventricular models reconstructed from late gadolinium-enhanced magnetic resonance imaging scans. We comprehensively characterized all possible pre-ablation and post-ablation VTs in simulations conducted with either an “average human VT”-based electrophysiological representation (i.e., EP_avg_) or with ±10% APD or CV (i.e., EP_var_); additional simulations were also executed in some models for an extended range of these parameters. The results showed that: (1) a subset of reentries (76.2–100%, depending on EP parameter set) conducted with ±10% APD/CV was observed in approximately the same locations as reentries observed in EP_avg_ cases; (2) emergent VTs could be induced sometimes after ablation in EP_avg_ models, and these emergent VTs often corresponded to the pre-ablation reentries in simulations with EP_var_ parameter sets. These findings demonstrate that the VT ablation target uncertainty in patient-specific ventricular models with an average representation of VT-remodeled electrophysiology is relatively low and the ablation targets stable, as the localization of the induced VTs was primarily driven by the remodeled structural substrate. Thus, personalized ventricular modeling with an average representation of infarct-remodeled electrophysiology may uncover most targets for VT ablation.

## Introduction

Ventricular tachycardia (VT), a life-threatening fast heart rhythm, occurs frequently in patients with myocardial infarction, and leading to sudden cardiac death. Although increased utilization of pre-procedural imaging (Fernandez-Armenta et al., 2013), high-density mapping (Anter et al., 2016), epicardial access (d’Avila et al., 2006), and more extensive ablation strategies (Di Biase et al., 2012; Jais et al., 2012) have all contributed to improved procedure outcome, a high efficacy of treatment for VT in the electrophysiology (EP) laboratory has not yet been achieved. At present, catheter ablation is the most effective method to eliminate VT, however, it only has modest success, 50–88% (Aliot et al., 2009). This is the result of limitations in current techniques for mapping the electrical functioning of the heart and identifying the targets for VT ablation.

Computational modeling has emerged as a powerful platform for the non-invasive investigation of lethal heart rhythm disorders and their treatment (Behradfar et al., 2014; Pathmanathan and Gray, 2014; Grandi and Maleckar, 2016; Loewe et al., 2018; Roney et al., 2018a,b); it has been used for risk stratification in patients with myocardial infarction (MI) (Vadakkumpadan et al., 2014; Arevalo et al., 2016; Deng et al., 2016) and prediction of reentry location (Ashikaga et al., 2013; Deng et al., 2015; Zahid et al., 2016). Computational technology has also been recently developed to guide patient ablation (the Virtual-heart Arrhythmia Ablation Targeting, or VAAT), and even used prospectively, as a prove of feasibility of the approach, in a small number of patients (Prakosa et al., 2018). In these models, the personalized element is limited to information that is extracted from non-invasive late gadolinium-enhanced magnetic resonance imaging (LGE-MRI) scans, which is the patient-specific ventricular geometry, and the spatial distribution of infarcted-remodeled tissue. Cell- and tissue scale EP in these models, while reflecting the fact that the substrate in VT patients involves EP ventricular remodeling (Dangman et al., 1982; Dun et al., 2004; Mendonca Costa et al., 2018), is derived from an average human set of EP parameters (i.e., EP_avg_) to represent non-infarcted tissue and remodeled gray zone (GZ). Clearly, an ideal option would be to use personalized EP in each model, however acquisition of such information is invasive, in contrast to the VAAT approach, which offers a non-invasive prediction of the ablation targets. Thus, the ablation targets that are predicted to terminate VT in post-MI patients using the VAAT approach might have a level of uncertainty associated with the fact that pre-determined EP is used.

Similar issues had previously arisen in using patient-specific models of persistent atrial fibrillation (AF) in patients with fibrotic remodeling (Trayanova, 2014; Zahid et al., 2016; Deng et al., 2017; Hakim et al., 2018). As the intention is that such models be used in the future to predict the patient-specific targets for AF, in a series of papers we sought to assess the level of uncertainty in such predictions (Deng et al., 2017; Hakim et al., 2018). Research in these studies demonstrated that while not always the same ablation targets are predicted under the examined variable EP conditions (Deng et al., 2017), the majority of the targets that were not predicted under the average EP conditions manifested themselves upon repeating the pacing protocol post-ablation (Hakim et al., 2018).

While persistent AF in the fibrotic substrate and VT post-MI have very different arrhythmia dynamics, they both result from a combination of remodeled structural substrate interacting with a remodeled EP. Thus, in this study, we aimed to systematically assess the effect of changes in the electrophysiological parameter set on the induced VTs and predicted ablation targets in personalized models of post-MI patient hearts.

## Materials and Methods

### Post-infarction Human Heart Models

For this retrospective study, we used data from 5 patients who were referred for catheter ablation of VT; their MRI data are used for model reconstruction. Details regarding patient information and model construction can be found in our previous publication (Prakosa et al., 2018). Briefly, the myocardium boundaries were semi-automatically segmented and regions of gray zone (GZ), core scar, and non-infarcted tissue were segmented from LGE-MRI by means of signal thresholding (Arevalo et al., 2016; Deng et al., 2016). Tetrahedral meshes were constructed directly from the segmented images using a previously described approach (Prassl et al., 2009), which uses the dual mesh of an octree applied directly to the segmented 3D image stacks. Fiber orientations in the mesh were assigned using a previously validated rule-based method (Bayer et al., 2012). In order to be able to conduct this research in a tractable manner, as it involves a very large number of simulations (see below), we conducted simulations in the left ventricle (LV) only in all these 5 models. The rationale for this choice is based on the fact that human infarcts resulting in VT are typically located in the LV (Cano et al., 2017; Martin et al., 2018); the LV was also the location where all the clinical VTs in these patients were sustained.

### Modeling Cell- and Tissue-Scale Variability of Ventricle

Electrophysiology properties were assigned in the model as previously described (Prakosa et al., 2018). Briefly, the mathematical description of electrical conduction in cardiac tissue was based on the monodomain representation (Plank et al., 2008). Core scar was modeled as passive tissue. Non-infarcted tissue and GZ were assigned human ventricular myocyte action potential dynamics (ten Tusscher et al., 2004). The values of the non-infarcted tissue conductivities used in this study were 0.255 and 0.0775 S/m in the longitudinal and transverse directions, respectively. Tissue in the GZ region was characterized with a 90% decrease in transverse conductivity to reflect connexin-43 remodeling in the infarct border zone (Yao et al., 2003).

**FIGURE 1:**
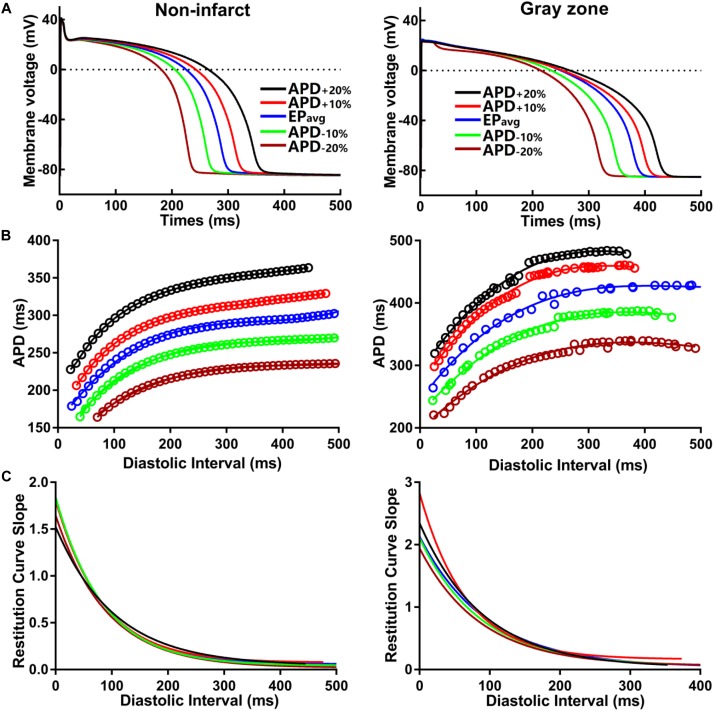
**(A)** Action potential (AP) traces for simulated normal (non-infarcted) (left) and GZ (right) ventricular myocytes, paced to steady-state (1000 stimuli at basic CL = 600 ms) under average human VT electrophysiology (EP_avg_) conditions, and with AP duration (APD_90_) variability (±10% and ±20%). **(B)** APD restitution relationships for the respective cell types shown in A. Fit lines obtained via exponential regression. **(C)** Plots showing APD restitution curve slope values for different diastolic intervals.

To establish baseline reentry dynamics in the absence of variability in the electrophysiological component of the infarcted substrate, we first conducted simulations under average human VT EP, as in previous studies (Arevalo et al., 2016; Deng et al., 2016). For brevity, these conditions are referred to as EP_avg_. We then ran simulations in ventricular models with the same geometric structure and infarcted tissue distribution to assess the effects of APD and CV variability. For simulations with APD variability, specific ionic currents were modified to achieve ±10% APD (Figure 1A,B), while minimally altering other AP properties, including resting membrane potential [V_m_], AP amplitude, and restitution curve slope (Figure 1C). To simplify ionic current parameters selection process, I_Kr_ and I_Ks_ were modified simultaneously in 10% increments from −90 to 300%, and the current values that resulted in APD closest to the target APD (i.e., ±10% or ±20% APD) in each case were used in this study (Table 1). For simulations with CV variability, the longitudinal and transverse components of the conductivity tensor were modified to achieve ±10% CV. To avoid biasing effects from the complex infarcted structure in ventricular models, CV values were calibrated by simulating wavefront propagation following stimulation of the center point in a slab model (6 cm × 6 cm × 2.7 mm) with uniform fiber orientations, and conductivity tensor values were adjusted until the desired longitudinal and transverse CVs were observed. In both families of cases (i.e., APD_±10%_ and CV_±10%_ conditions), parameter changes required to achieve the desired emergent property were different for non-infarcted and GZ tissues. Complete details about these changes and the resulting variability in APD and CV are presented in Tables 1, 2, respectively.

**Table 1:** Cell-scale electrophysiological model parameters modified to achieve ±10% and ±20% action potential duration (APD) in non-infarcted and GZ tissues.

		Parameters changed		APD_target_ (ms)	APD_actual_ (ms)	Percentage of APD changed	APA (mV)	V_rest_ (mV)
Non-infarcted	APD_−20%_	+80% I_Kr_	+80% I_Ks_	230.8	233.28	−20.8%	126.3	−84.8
	APD_−10%_	+30% I_Kr_	+30% I_Ks_	263.8	262.44	−9.5%	126.1	−84.8
	EP_avg_	…	…	291.6	291.6	…	126.0	−84.7
	APD_+10%_	−20% I_Kr_	−20% I_Ks_	315.9	320.76	8.3%	126.0	−84.7
	APD_+20%_	−40% I_Kr_	−40% I_Ks_	348.5	349.92	19.5%	126.0	−84.6
Gray zone	APD_−20%_	+80% I_Kr_	+80% I_Ks_	309.7	306.24	−19.1%	110.1	−85.3
	APD_−10%_	+30% I_Kr_	+30% I_Ks_	348.1	344.52	−9.1%	109.9	−85.3
	EP_avg_	…	…	382.8	382.8	…	109.9	−85.2
	APD_+10%_	−20% I_Kr_	−20% I_Ks_	409.4	421.08	7.0%	109.3	−85.2
	APD_+20%_	−30% I_Kr_	−30% I_Ks_	454.2	459.36	18.7%	108.7	−85.1

*“…” indicates no change. EP_avg_, average human VT electrophysiology; APA, action potential amplitude; V_rest_, resting membrane potential; I_Ks_, slow delayed rectifier potassium current; I_Kr_, rapid delayed rectifier potassium current. APD_90_ was used to calculate APD. APD_target_: The target APD (±10% or ±20% APD). APD_actual_: It represents the actual value of APD (closest to the target) obtained by altering I_Kr_ and I_Ks_ simultaneously (see section “Materials and Methods).*

**Table 2:** Tissue-scale electrophysiological model parameters modified to achieve ±10% and ±25% longitudinal and transverse conduction velocities (*CV*_L_ and *CV*_T_, respectively) in non-fibrotic and fibrotic atrial tissues.

		Parameters changed		CV_L_ (cm/s)	CV_T_ (cm/s)	CV_L_:CV_T_
Non-infarcted	CV_−25%_	−47.6% σ_iL_	−47.6% σ_iT_	41.1	26.2	1.57
	CV_−10%_	−22.2% σ_iL_	−22.1% σ_iT_	49.3	30.7	1.61
	EP_avg_	…	…	54.8	33.5	1.63
	CV_+10%_	+28.5% σ_iL_	+28.1% σ_iT_	60.3	36.6	1.65
	CV_+25%_	+86.4% σ_iL_	+86.5% σ_iT_	68.5	41.3	1.66
Gray zone	CV_−25%_	−46.1% σ_iL_	−46.1% σ_iT_	32.6	15.3	2.13
	CV_−10%_	−21.4% σ_iL_	−21.3% σ_iT_	39.1	17.5	2.23
	EP_avg_	…	…	43.4	18.8	2.42
	CV_+10%_	+25.6% σ_iL_	+25.5% σ_iT_	47.7	20.0	2.39
	CV_+25%_	+81.2% σ_iL_	+81.4% σ_iT_	54.6	21.9	2.49

*“…” indicates no change. σ_iL_ and σ_iT_, longitudinal and transverse conductivity tensor values; EP_avg_, average human VT electrophysiology.*

As experimental measurements (Britton et al., 2013) have shown that APD and CV could vary in a larger range, we conducted additional simulations in two models only (for computational tractability) with APD_±20%_ and CV_±25%_, and in one model, with a combination of APD_±20%_ and CV_±25%_. The parameter changes were listed in Tables 1, 2. We chose APD_±20%_ instead of APD_±25%_ because APD_+25%_ resulted in longer APD than the upper limit of APD in experimental measurements (364 ms vs. 340 ms) (Britton et al., 2013).

### Stimulation Protocol

All simulations were performed using the software package CARP^1^ on a parallel computing platform (Vigmond et al., 2003). A programmed electrical stimulation protocol similar to standard clinical stimulation protocols was performed to examine the arrhythmogenic propensity of the post-MI ventricular models (Cheng et al., 2013). All models were paced from 7 locations, optimized for maximum likelihood of VT induction, for 6 beats (S1) at a cycle length (CL) of 600 ms followed by a premature stimulus (S2) initially given at 90% of S1 CL. The timing between S1 and S2 was progressively shortened until reentry was induced. If reentry was not induced, a second premature stimulus (S3) was delivered after S2. If VT was still not induced, a third premature stimulus (S4) was delivered after S3. Three seconds of VT were simulated.

The smallest S1-S2 coupling interval was determined in the fowling way: Six stimulus of S1 (CL = 600 ms) were delivered; then S2 was given at 250 ms. If excitation was elicited, then we decreased S2 to 200 ms. If the excitation could propagate again, then we decreased S2 in 10 ms intervals until excitation could not be elicited. The S2 coupling interval was the last one that elicited excitation propagation. The same protocol was applied to determine the smallest S2-S3 and S3-S4 intervals. If reentry was induced after the S2 stimulus, then no addition stimulus was given.

### Ablation Strategy

First, the reentrant circuit was identified in all models with EP_avg_ through analysis of the 3D reentrant wave propagation. *In silico* ablation was performed by rendering the tissue in the narrowest region of the reentrant circuit [typically a channel in the scar as in (Prakosa et al., 2018)] non-excitable. Each virtual ablation lesion was of radius in our simulation was 3.5 mm, which is similar to the clinical catheter size (Narayan et al., 2012). The VT induction protocol was then repeated to establish that VT was no longer inducible in the ablated LV substrate. If a new VT arose in the post-ablation model, then the new VT circuit was analyzed and additional ablation targets were determined and implemented in the model, and the pacing protocol was repeated until VT was no longer inducible.

All simulations were then repeated in the family of models with EP_var_ (APD_±10%_ and CV_±10%_, 20 additional 3D LV models; 25 3D LV models in total for this study). For VTs induced in each of these models, if the reentry morphology was the same or similar to VT induced in the corresponding EP_avg_ model, the ablation lesion from the EP_avg_ model was applied to the same location in the EP_var_ model. By similar reentry morphology we refer to reentries that have the same wavefront conduction pathway, with the same critical conduction channels (or conduction isthmus), but could have different CL, and even different reentry direction. If VT in the EP_var_ model was terminated, the ablation lesions in EP_var_ and EP_avg_ models were considered “overlapping.” If VT persisted in the EP_var_ model despite the ablation, then these two VTs were considered non-overlapping. Then ablation in the EP_avg_ model proceeded as described above. Finally, the number of ablation lesions was compared between EP_avg_ and EP_var_ models.

**Table 3:** Summary of results for simulations conducted in EP_avg_ models.

ID	# reentries pre-ablation	# reentries post-ablation	Extent of ablated tissue (%)
P01	4	1	1.3
P02	3	0	0.2
P03	2	0	0.1
P04	4	0	2.1
P05	3	0	0.9

## Results

For simulations conducted under EP_avg_ conditions, rapid pacing induced sustained VTs in all 5 patient-specific ventricular models. Table 3 summarized the results of simulations conducted in EP_avg_ models. A total of 16 distinct reentries were induced in five models under the EP_avg_ condition, and after ablation, only one new distinct reentry was induced. The average amount of ablated ventricular tissue under EP_avg_ condition was 0.92%. Before ablation, there were 2–4 different sustained VTs (3.2 ± 0.8) induced in each model, and the organizing centers of all 16 VTs perpetuated in distinct locations. Ablation, as described in Section “Materials and Methods,” did not always render the remodeled substrate non-inducible for VT; in some cases, new VTs could arise. Specifically, after ablation, 4 patient models did not have emergent VTs, while 1 patient model had one emergent VT at a location different from those before ablation. The volume of the simulated ablation lesions that terminated the VTs, both original and emergent, in each model was 0.92 ± 0.83% of the total ventricular volume (min: 0.1%, max: 2.1%).

**Table 4:** Summary of simulation results regarding the number of VTs obtained for the EP_avg_ and EP_var_ (APD_±10%_ and CV_±10%_) models with the 5 patient-specific ventricular geometries.

		Total number of VTs	Number of VTs in EP_avg_ models	Percentage of VTs in EP_var_ models that were also present in EP_avg_ (%) models (%)
# of induced VTs in EP_avg_	Pre- ablation	16	–	–
	Post-ablation	17	–	–
# of induced VTs in APD_+10%_	Pre- ablation	10	10	100
	Post-ablation	10	10	100
# of induced VTs in APD_−10%_	Pre- ablation	11	9	81.8
	Post-ablation	12	10	83.3
# of induced VTs in CV_+10%_	Pre- ablation	9	9	100
	Post-ablation	9	9	100
# of induced VTs in CV_−10%_	Pre- ablation	20	14	70
	Post-ablation	21	16	76.2
# of induced VTs in APD_+20%_^∗^	Pre- ablation	3	3	100
	Post-ablation	3	3	100
# of induced VTs in APD_−20%_^∗^	Pre- ablation	3	3	100
	Post-ablation	4	3	75
# of induced VTs in CV_+25%_^∗^	Pre- ablation	3	3	100
	Post-ablation	3	3	100
# of induced VTs in CV_−25%_^∗^	Pre- ablation	6	5	83.3
	Post-ablation	7	5	71.4
# of induced VTs in APD_+20%_ & CV_+25%_^∗∗^	Pre- ablation	0	0	0
	Post-ablation	0	0	0
# of induced VTs in APD_−20%_ & CV_+25%_^∗∗^	Pre- ablation	0	0	0
	Post-ablation	0	0	0
# of induced VTs in APD_+20%_ & CV_−25%_^∗∗^	Pre- ablation	1	1	100
	Post-ablation	1	1	100
# of induced VTs in APD_−20%_ & CV_−25%_^∗∗^	Pre- ablation	3	3	100
	Post-ablation	4	3	75

*“Number of VTs in EP_avg_ models” represents the number of reentry morphologies induced in each set of modified conditions with similar or same reentry morphologies induced under the EP_avg_ condition. The last column in Table 4 is calculated as “Number of VTs in EPavg models”/” Total number of VTs”, which represents how many reentry morphologies induced under each of the modified condition corresponded to the reentry morphologies induced under EP_avg_. ^∗^, only 2 models were used with this EP parameter set; ^∗∗^, only 1 model was used with this EP parameter set.*

**FIGURE 2:**
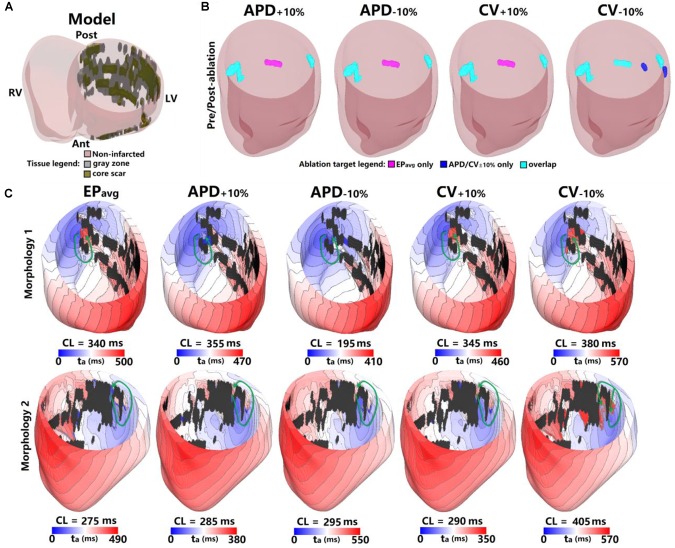
Geometry and simulated activation maps and ablation lesions under average human VT EP_avg_ and variable APD/CV conditions for model 5. **(A)** Geometric model of the infarcted heart of patient model 5. **(B)** Ablation lesions for simulations with the 5 different parameter sets. **(C)** Highlighting two VT morphologies in which the same pacing sequence applied in the same model led to the initiation of VT driven by an RD in the same ventricular region, regardless of the variability in APD/CV. t_a_, activation time. The black areas in panel C for all figures are core scar – there is no electrical activation there.

**FIGURE 3:**
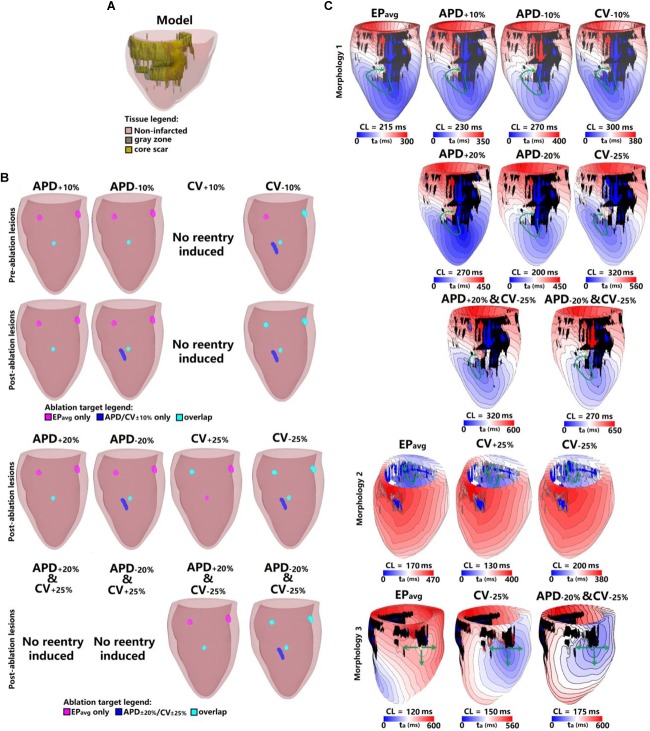
Geometry and simulated activation maps and ablation lesions under average human VT EP_avg_ and variable APD/CV conditions for model 2. **(A)** Geometric model of the infarcted heart of patient model 2. **(B)** Ablation lesions for simulations for the 13 different parameter sets. **(C)** Highlighting one VT morphology in which the same pacing sequence applied in the same model led to the initiation of VT driven by an RD in the same ventricular region, regardless of the variability in APD/CV. t_a_, activation time. The black areas in panel C for all figures represent core scar – there is no electrical activation there.

Comprehensive summary data for the VTs induced in simulations conducted in all 5 ventricular models under the 5 different APD/CV_±10%_ conditions each (EP_avg_, APD_±10%_, and CV_±10%_) are provided in Table 4 and Supplementary Tables S1–S4. Of all 25 3D LV models used in this study, VT could be induced in 24. The only model in which VT could not be induced is that of patient 2 with CV_+10%_ parameters. In terms of the total number of VTs, 22 unique VTs were induced in the 24 inducible models. Eight of the VTs were induced under all five electrophysiological conditions; these were for patients 1, 3, 4, and 5. The activation maps of these 8 VTs are shown in Figure 2 and Supplementary Figures S1–S3. The VT in model 2 that was induced under 4 of the 5 EP parameter sets (and not under the CV_+10_) is shown in Figure 3. Of the 4 varied parameter sets (APD_±10%_ and CV_±10%_), APD_±10%_, and CV_+10%_ resulted in less VTs, while CV_−10%_ resulted in more VTs compared with the EP_avg_ conditions.

There were 10 distinct reentries induced in models with the APD_+10%_ parameter set before ablation, and all of them corresponded to VTs under EP_avg_ conditions (Table 4 and Supplementary Table S1). There were no emergent reentries after ablation under these electrophysiological conditions. For the CV_+10%_ parameter set (Table 4 and Supplementary Table S3), there were 9 distinct VTs induced before ablation, and they all also matched the VTs in EP_avg._ Under the APD_−10%_ conditions (Table 4 and Supplementary Table S2), there were 11 distinct VTs before ablation, and 9 of them corresponded to those under EP_avg_ conditions. Only for patient 2 there was one emergent VT after ablation, which was different from any VT under EP_avg_ conditions before and after ablation (the blue lesion in Panel B of Figure 3). In patient 1, two reentries were new and different from any VT under EP_avg_ conditions before ablation, but one of them was induced under EP_avg_ conditions after ablation (Panel B in Supplementary Figure S1). Thus overall, for the APD_−10%_ parameter set, 10 out of 12 VTs had matching reentries under EP_avg_ conditions, while the remaining 2 VTs were new, and different from the reentries in EP_avg_.

For the CV_−10%_ parameter set (Table 4 and Supplementary Table S4), there were 20 distinct VTs induced before ablation, and 14 of them had corresponding reentries under EP_avg_ conditions. After ablation, there was only one emergent VT in patient 2 (Panel B in Figure 3), but 2 of the VTs induced before ablation were also induced under EP_avg_ conditions, so 16 out of 21 VTs in the CV_−10%_ parameter set models had matching VTs under EP_avg_ after ablation.

The results for one example model (patient 5) under all 5 EP parameter sets are shown in Figure 2. There were 3 distinct VTs induced under EP_avg_, one located at the septum (Figure 2C, Morphology 1 in EP_avg_), another one at the LV lateral wall (Figure 2C, Morphology 2 in EP_avg_), and the third one located at the posterior wall (morphology not shown). For the APD_±10%_ and CV_±10%_ parameter sets, both resulted in two reentries being induced, which were located at the septum and the LV lateral wall. Under CV_−10%_ conditions, there were 5 distinct VTs induced, and 3 of them had matching reentries in EP_avg_ models (ablation lesions in Panel B of Figure 2).

For the 2 VTs induced under all 5 conditions, the morphology and location were very similar for across all models. For morphology 1 (first row in Figure 2C), the reentry was circular, with the only difference being the CL, which varied from 195 ms to 380 ms. After ablating the narrowest channel region in VT morphology 1, the reentry was terminated. After applying the ablation lesions from EP_avg_ to the other 4 conditions (light blue lesions on the left side of Figure 2B), and the reentry at that region was terminated in all 4 conditions too. For morphology 2, the reentry was circular in all 5 conditions, but the conduction pathway in CV_−10%_ condition showed differences. Under EP_avg_, APD_±10%_ and CV_+10%_ conditions, there was only one conduction pathway, but under the CV_−10%_ condition, the reentry had two condition pathways, one of which, the one close to posterior wall, was the same under the other 4 conditions. After ablating the narrowest channel region in morphology 2 in EP_avg_, the reentry was terminated. Then applying the ablation lesion from EP_avg_ to the four other EP versions (light blue lesions on the right side of Figure 2B), the reentry under APD_±10%_, and CV_+10%_ conditions was terminated. But applying the ablation lesions from EP_avg_ didn’t terminate the reentry induced in CV_−10%._ After ablating the narrowest parts of both conducting pathways under CV_−10%_ conditions, reentry was terminated (the light blue and blue on the right-most side of CV_−10%_ in Figure 2B). The VTs induced at the posterior wall in EP_avg_ could not be induced under APD_±10%_ and CV_+10%_ conditions; the ablation lesion for that reentry is shown in purple in the first 3 panels of Figure 2B. The 3 VTs induced in EP_avg_ were all induced in CV_−10%_ (light blue lesions in CV_−10%_ panel of Figure 2B), but there were 2 new VTs induced in CV_−10%_, of which the 2 ablation lesions are shown as blue in the CV_−10%_ panel in Figure 2B.

**FIGURE 4:**
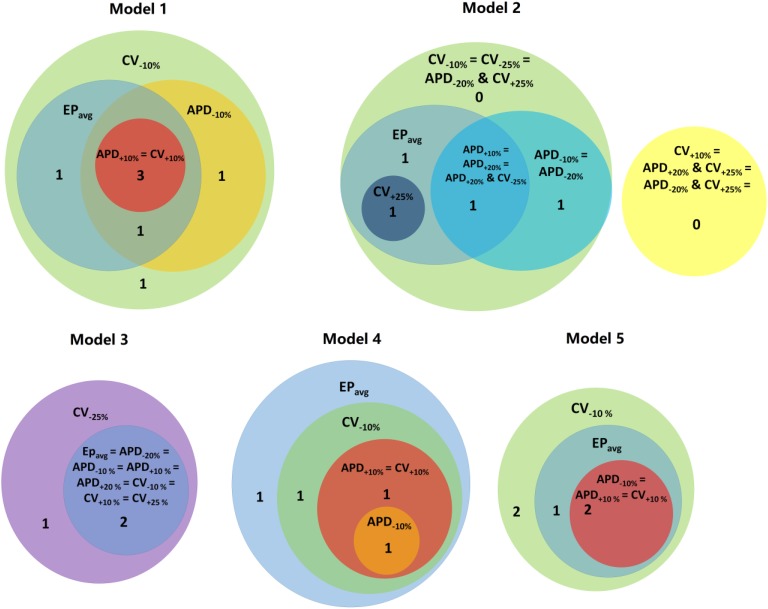
Venn diagrams for each models under average human VT EP_avg_ and variable APD/CV conditions. Numbers represent the number of distinct reentries in each case.

Figure 3 shows the results for another patient model (patient 2) for the 5 EP parameter sets. There were 3 VTs induced in EP_avg_, with two of them being on the anterior wall, and the third on septum. For CV_+10%_, there was no reentry induced, and this is the only model variant of all 25 model variants in 5 the patients in which no reentry could be induced. Before ablation, the models with APD_±10%_ parameters had only one reentry induced, on the anterior middle wall (Figure 3C), and this reentry is similar to the one induced in EP_avg_ (first panel in Figure 3C). In CV_−10%,_ there were 3 VTs induced, and two of them had matching reentries in EP_avg_. But the one on the middle anterior wall had minor differences compared to the corresponding one in EP_avg_: it had 2 conduction pathways (CV_−10%_ panel in Figure 3C), with the pathway on the right matching the one in EP_avg_, but the left side was a new pathway. Applying the lesions determined from EP_avg_, the middle anterior wall VT could not be terminated in CV_−10%_ (light blue lesion in the CV_−10%_ panel of the first row in Figure 3B), and an additional lesion had to be applied to narrowest portion of the left condition pathway in CV_−10%_ to terminate the reentry on the anterior middle wall (blue lesion in the CV_−10%_ panel of the first row in Figure 3B). After ablation, no reentry could be induced in EP_avg_ and APD_+10%_, but in APD_−10%,_ a new VT emerged at the vicinity of the ablation lesion on the anterior wall; the condition pathway of this RD is very similar to the left side pathway of the VT induced in CV_−10%_. Applying the EP_avg_ ablation lesions to CV_−10%_ terminated the VT. In CV_−10%_ condition, after ablation, there was an emergent VT, which matched the VT on the left side of the anterior wall in EP_avg_; applying the ablation lesion from EP_avg_ terminated the emergent reentry in CV_−10%._ Results of the study for the other 3 patients are shown in Supplementary Figures S1–S3. Vann diagrams summarizing the results of simulations for all above cases are presented in Figure 4.

Next, we present results of simulations with the additional EP parameter sets (from the extended parameter range) in the limited number of models, as described in Section “Materials and Methods.” Results from simulations with model 2 are presented in Figure 3. Upon change of APD from +10% to +20% and from −10 to −20%, there was no difference in reentry morphology and reentry location. When changing CV from ±10 to ±25%, there was a difference between results for the CV_+10%_ and CV_+25%_ cases: no reentry was induced in CV_+10%_ simulations, and there was one reentry induced in CV_+25%_. There was no difference between the cases of CV_−10%_ and CV_−25%_. Results for model 3 and the same parameter range are presented in Supplementary Figure S2. For this model, there was no difference between results for the APD_±20%_ and CV_+25%_ conditions, but under the CV_−25%_ condition, one new reentry emerged (panel D of Supplementary Figure S2) that was not present for the other modified APD and CV cases. Thus, based on the simulation results for the 2 patients with APD_±20%_ and CV_±25%_, we conclude that APD changes within the range of ±20% had no additional effects on reentry location. Changing CV in the range (±25%) resulted in changes in reentry location only when CV was at the boundaries of that range. A Venn diagram of these results is provided in Figure 4.

Finally we executed simulations in one model with a combination of parameter changes, as described in Section “Materials and Methods.” Conducting simulations with model 2, the combinations of APD_+20%_ & CV_+25%_ and APD_−20%_ & CV_+25%_ conditions did not result in reentry. There were two reentries induced under the APD_+20%_ & CV_−25%_ condition, one of which corresponded to the reentry morphology in EP_avg_ (see Figure 3), and the other one was a new VT morphology with respect to the EP_avg_ case. There were four VT morphologies induced in the APD_−20%_ & CV_−25%_ condition, 3 of which corresponded to EP_avg_, and one was new. The combined reduction/increase of CV and APD produced only one different VT morphology as compared to EP_avg_. This VT morphology was the same as the ones induced for cases where only APD or only CV was changed. Thus we conclude, based on the simulations in model 2, that combined reduction/increase of CV and APD within the range of ±20% or ±25% had no additional effects on VTs compared with only changing APD or CV.

## Discussion

In this study, we used computational models reconstructed from LGE-MRI scans of the infarcted ventricle of individuals with VT to assess the sensitivity of VT localization to variability in cell- and tissue-scale electrophysiological parameters. We showed that: (1) in simulations conducted with ±10% APD/CV, a subset of VTs (76.2–100%, depending on the EP parameter set) were observed in approximately the same locations as those in the EP_avg_ case; (2) EP_avg_ conditions resulted in more VTs (5–8) than in APD_±10%_ and CV_+10%_, and in less reentries than in CV_−10%_; (3) Emergent VTs were induced sometimes post-ablation, and the emergent reentries often matched the pre-ablation VTs in models with other parameter sets. (4) Where the VTs were robust to APD/CV variability, there were differences, as expected, in macroscopic arrhythmia features such as CL and total activation time.

About 53% (9/17) of the reentries that occurred in EP_avg_ were also induced in all other four parameter set models (±10% APD/CV), and the remainder of reentries were induced in at least one of the four parameter set models. Interestingly, all the reentries induced in APD_+10%_ and CV_+10%_ models had matching reentries in the EP_avg_ models. The models with EP_avg_ were more inducible than the APD_+10%_ and CV_+10%_ models, as the wavelength in the former case was smaller, allowing for more space for the reentry to propagate and be sustained. While there were new VTs induced in models with APD_−10%_ and CV_−10%_, more than 76% of the VTs occurred also in models with EP_avg_. The models with APD_−10%_ had a smaller number of induced VTs than the corresponding EP_avg_ models. A possible explanation for this finding is that APD in the EP_avg_ parameter set falls in the arrhythmogenesis susceptibility window of APD (Clayton and Holden, 2005), where there is a unidirectional block of electrical conduction through a VT-sustaining channel. Upon further reduction of APD, the VT channel conducts bi-directionally, resulting in a smaller number of included reentries.

In the additional simulations with the extended range of APD and CV and their combination, the results with the combined reduction/increase of CV and APD were very similar, in terms of reentry location, and pathway to those when varying APD or CV independently. Furthermore, APD_±20%_ and CV_±25%_ cases had similar outcomes to those with APD_±10%_ and CV_±10%_. Thus, simulating only ±10% APD or ±10% CV changes may be sufficient in representing the results from simulations with larger electrophysiological parameters variations.

These findings present evidence that VT localization is fairly robust to electrophysiological variability, and that the distribution of the infarcted tissue (core scar and GZ) might play a dominant role in determining the location of infarct-related VTs. Our results suggest that EP_avg_ models identified most of the VT ablation targets that were consistently observed under different EP conditions. Nonetheless, there was a small subset of reentries that were observed only under EP_avg_ simulations. Furthermore, in some models, VTs only emerged upon APD and/or CV change, suggesting that there could be a small number of potential ablation targets that may not be revealed using the EP_avg_ parameter set. Furthermore, for reentries with the same morphology and location of the critical conduction isthmus (and thus the same ablation target) we observed differences in CL, total activation time and in rare cases, even direction of propagation. Finally, we demonstrated that majority of ablation targets that were not predicted under EP_avg_ conditions manifested themselves upon repeating the pacing protocol post-ablation, similar to the findings in modeling of ablation for persistent AF in patients with fibrotic remodeling (Hakim et al., 2018).

The results shown in this paper differ from our atrial sensitivity analysis study (Deng et al., 2017) because the mechanism of reentry in VT and AF are different. In the atrial models, there is only non-fibrotic and fibrotic tissue (both are excitable tissues, but fibrotic tissue has modified EP). In contrast, in the ventricular models, there is additionally a core scar. Thus reentries in the atrial models had a larger functional component and their morphologies and locations could be affected, by somewhat larger degree, by changes in APD or CV. In infarct-related VT, most of the reentries were anchored to core scar or included conduction through channels, consistent with published data (Martin et al., 2018).

Patient-specific computational modeling of VT has been proposed as a new approach to non-invasively predict personalized VT ablation targets in post-MI patients (Prakosa et al., 2018). The present study demonstrates that simulations conducted under EP_avg_ conditions identified most of VTs that were consistently observed under multiple different EP conditions. The uncertainty in the post-MI VT ablation targets under EP_avg_ is further mitigated by the design of the pipeline for determining the optimal set of targets [as in the VAAT approach (Prakosa et al., 2018)], where the protocol is repeated post-ablation with virtual targets incorporated, until the remodeled substrate is no longer capable of sustaining VT. This allows the protocol to reveal additional VTs under EP_avg_ that are not manifested following the initial pacing protocol prior to virtual ablation, but appear right away in simulations using models with different EP properties.

## Conclusion

In conclusion, we showed that perturbing APD and CV by ±10% caused relatively small variation in VT localization. In a small number of cases, new reentries at locations distinct from those in EP_avg_ emerged when EP parameters changed. Most of those were revealed in EP_avg_ models when the simulation protocol for determining the ablation targets was repeated with the initial targets incorporated in the models. Overall, the localization of the induced VTs was primarily driven by the remodeled structural substrate. Thus, personalized ventricular modeling with an average representation of infarct-remodeled EP may uncover most targets for VT ablation.

## Limitations

The first limitation is that we only considered a relatively limited subset of the parameter space of four discrete changes (±10% APD and ±10% CV). Considering the relatively low CV and long APD in GZ used in our model, further changes in these parameters will make these values fall out of the experimental range. Furthermore, we conducted a large number of simulations with 25 different 3D LV models. The sheer amount of computational time makes exploration of additional ranges of parameter values technically difficult. The second limitation in this study is that in order to achieve different APD values, we only changed I_Kr_ and I_Ks_ currents in our single cell models. We did not alter the Ca current as it would not only affect APD restitution, but would also have impact on other single cell properties and thus making the organ scale modeling too complicated to analyze. Furthermore, as the goal of this study was to provide a sensitivity analysis of ablation targeting, we did not explore the mechanisms of all the observed phenomena, including arrhythmogenesis arising from locally reduced APD. Finally, our model did not consider the influence of Purkinje system, the role of the right ventricle, and potential alterations in fiber orientation that cannot be captures by our patient-specific rule-based approach.

## Author Contributions

DD, AP, and PN performed the LGE-MRI scan segmentation and model creation. DD performed the simulations of VT in all models. DD, AP, and NT analyzed the data. DD, JS, and NT wrote the manuscript. All authors discussed the results and commented on the manuscript.

## Conflict of Interest Statement

The authors declare that the research was conducted in the absence of any commercial or financial relationships that could be construed as a potential conflict of interest.

## Footnotes

1 https://carpentry.medunigraz.at/carputils/
