# Supplementary material for: Sensitivity of Ablation Targets Prediction to Electrophysiological Parameter Variability in Image-Based Computational Models of Ventricular Tachycardia in Post-infarction Patients
Source: Front Physiol. 2019 May 24;10:628. doi: 10.3389/fphys.2019.00628 (PMC6543853; doi:10.3389/fphys.2019.00628)

Table S1. Summary of simulation results regarding reentries obtained from EP_{avg} and $APD_{+10\%}$ simulations in 5 patient-specific ventricular models.

	# of overlapping VT ablation lesions in EP_{avg} and $APD_{+10\%}$ models		# of induced VTs in EP_{avg}		# of induced VTs in $APD_{+10\%}$	
	Pre- Ablation	*Post-Ablation*	*Pre- Ablation*	*Post-Ablation*	*Pre- Ablation*	*Post-Ablation*
P01	3	3	4	1	3	0
P02	1	1	3	0	1	0
P03	2	2	2	0	2	0
P04	2	2	4	0	2	0
P05	2	2	3	0	2	0
Total VTs	10	10	16	17	10	10
VT overlap (%)	-	-	10/16 (62.5%)	10/17 (58.8%)	10/10 (100%)	10/10 (100%)

The total number of VTs in Post-Ablation EP_{avg} and $APD_{+10\%}$ models is the sum of number of VTs in pre-ablation models and the new emergent VT in post-ablation models.

Table S2. Summary of simulation results regarding reentries obtained from EP_{avg} and $APD_{-10\%}$ simulations in 5 patient-specific ventricular models.

	# of overlapping VT ablation lesions in EP_{avg} and $APD_{-10\%}$ models		# of induced VTs in EP_{avg}		# of induced VTs in $APD_{-10\%}$	
	Pre-Ablation	*Post-Ablation*	*Pre-Ablation*	*Post-Ablation*	*Pre- Ablation*	*Post-Ablation*
P01	3	4	4	1	5	0
P02	1	1	3	0	1	1
P03	2	2	2	0	2	0
P04	1	1	4	0	1	0
P05	2	2	3	0	2	0
Total VTs	9	10	16	17	11	12
VT overlap (%)	-	-	9/16 (56.3%)	10/17 (58.8%)	9/11 (81.8%)	10/12 (83.3%)

The total number of VTs in Post-Ablation EP_{avg} and $APD_{-10\%}$ models is the sum of number of VTs in pre-ablation models and the new emergent VT in post-ablation models.

Table S3. Summary of simulation results regarding reentries obtained from EP_{avg} and $CV_{+10\%}$ simulations in 5 patient-specific ventricular models.

	# of overlapping VT ablation lesions in EP_{avg} and $CV_{+10\%}$ models		# of induced VTs in EP_{avg}		# of induced VTs in $CV_{+10\%}$	
	Pre- Ablation	*Post-Ablation*	*Pre- Ablation*	*Pre- Ablation*	*Post-Ablation*	*Pre- Ablation*
P01	3	3	4	1	3	0
P02	0	0	3	0	0	0
P03	2	2	2	0	2	0
P04	2	2	4	0	2	0
P05	2	2	3	0	2	0
Total VTs	9	9	16	17	9	9
VT overlap (%)	-	-	9/16 (56.3%)	9/17 (52.9%)	9/9 (100%)	9/9 (100%)

The total number of VTs in Post-Ablation EP_{avg} and $CV_{+10\%}$ models is the sum of number of VTs in pre-ablation models and the new emergent VT in post-ablation models.

Table S4. Summary of simulation results regarding reentries obtained from EP_{avg} and $CV_{-10\%}$ simulations in 5 patient-specific ventricular models.

ID	# of overlapping VT ablation lesions in EP_{avg} and $CV_{-10\%}$ models		# of induced VTs in EP_{avg}		# of induced VTs in $CV_{-10\%}$	
	Pre- Ablation	*Post-Ablation*	*Pre- Ablation*	*Pre- Ablation*	*Post-Ablation*	*Pre- Ablation*
P01	4	5	4	1	7	0
P02	2	3	3	0	3	1
P03	2	2	2	0	2	0
P04	3	3	4	0	3	0
P05	3	3	3	0	5	0
Total VTs	14	16	16	17	20	21
VT overlap (%)	-	-	14/16 (87.5%)	16/17 (94.1%)	14/20 (70%)	16/21 (76.2%)

The total number of VTs in Post-Ablation EP_{avg} and $CV_{-10\%}$ models is the sum of number of VTs in pre-ablation models and the new emergent VT in post-ablation models.

Figure S1. Geometry and simulated activation maps and ablation lesions under average human VT electrophysiology (EP_{avg}) and variable APD/CV conditions for model 1. **(A)** Geometric model of the infarcted heart of patient model 1. **(B)** Ablation lesions for simulations for the 5 different parameter sets. **(C)** Highlighting three VT morphologies in which the same pacing sequence applied in the same model led to the initiation of VT driven by an RD in the same ventricular region, regardless of the variability in APD/CV. t_a: activation time. The black areas in panel C for all figures are core scar – there is no electrical activation there.

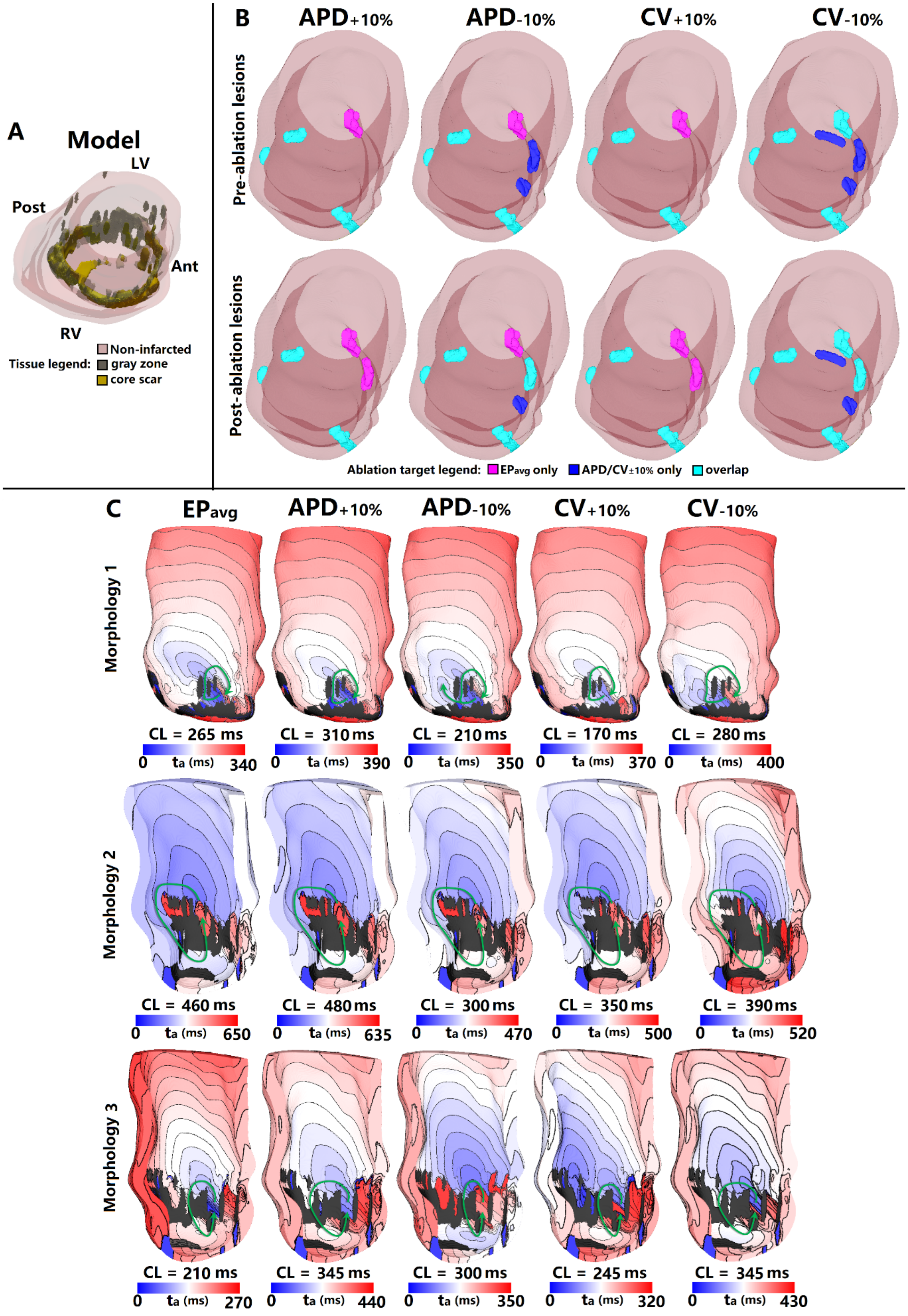

A
Model
LV
Post
Ant
RV
Tissue legend:
Non-infarcted
gray zone
core scar
B
APD+10%
APD-10%
CV+10%
CV-10%
Pre-ablation lesions
Post-ablation lesions
Ablation target legend:
EPavg only
APD/CV±10% only
overlap
C
EPavg
APD+10%
APD-10%
CV+10%
CV-10%
Morphology 1
CL = 265 ms
CL = 310 ms
CL = 210 ms
CL = 170 ms
CL = 280 ms
0 ta (ms) 340
0 ta (ms) 390
0 ta (ms) 350
0 ta (ms) 370
0 ta (ms) 400
Morphology 2
CL = 460 ms
CL = 480 ms
CL = 300 ms
CL = 350 ms
CL = 390 ms
0 ta (ms) 650
0 ta (ms) 635
0 ta (ms) 470
0 ta (ms) 500
0 ta (ms) 520
Morphology 3
CL = 210 ms
CL = 345 ms
CL = 300 ms
CL = 245 ms
CL = 345 ms
0 ta (ms) 270
0 ta (ms) 440
0 ta (ms) 350
0 ta (ms) 320
0 ta (ms) 430

Figure S2. Geometry and simulated activation maps and ablation lesions under average human VT electrophysiology (EP_{avg}) and variable APD/CV conditions for model 3. **(A)** Geometric model of the infarcted heart of patient model 3. **(B)** Ablation lesions for simulations for the 9 different parameter sets. **(C)** Highlighting two VT morphologies in which the same pacing sequence applied in the same model led to the initiation of VT driven by an RD in the same ventricular region, regardless of the variability in APD/CV. t_a: activation time. **(D)** One new unique VT morphology in $CV_{-25\%}$ condition. The black areas in panel C for all figures are core scar – there is no electrical activation there.

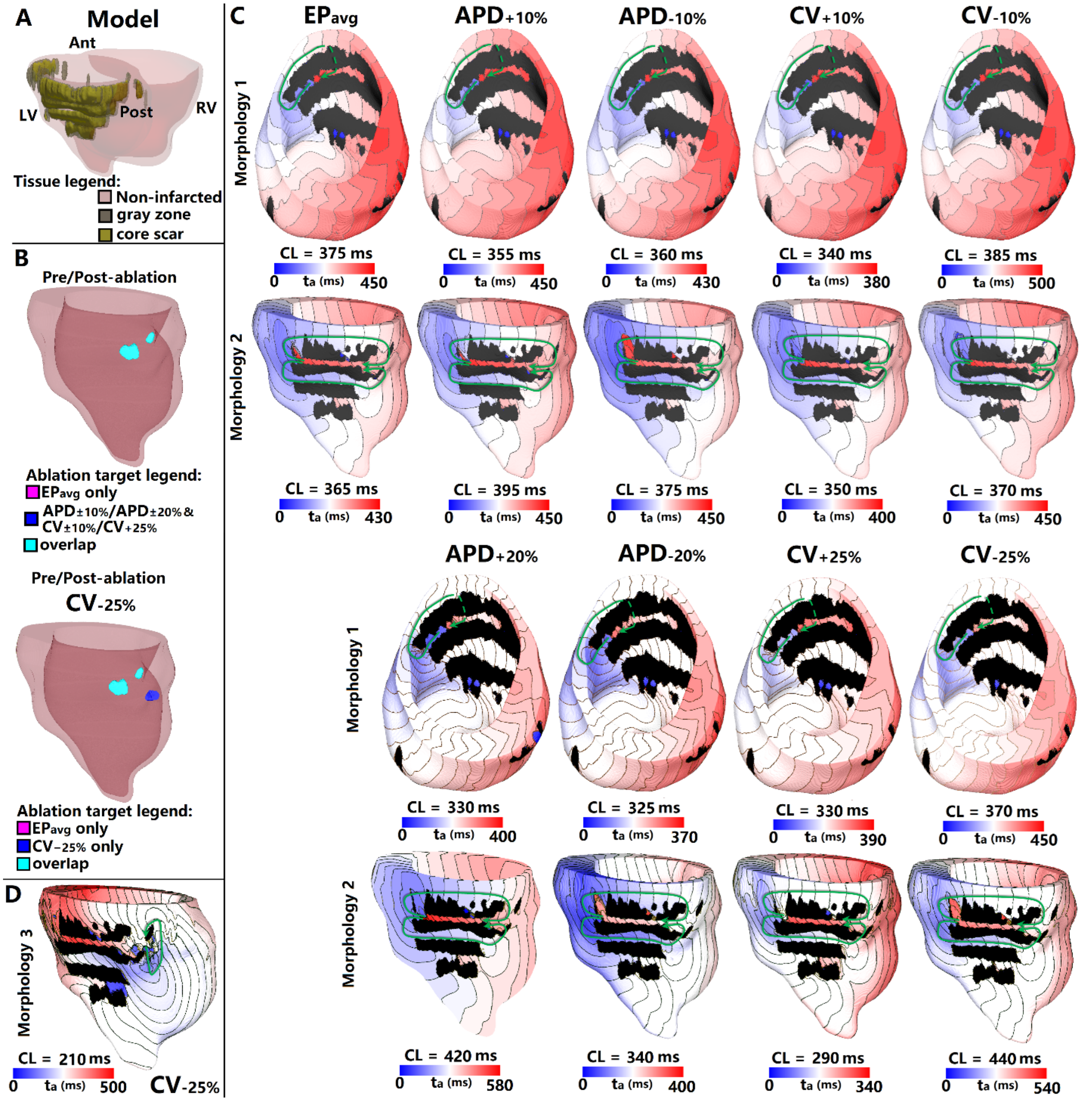

Figure S3. Geometry and simulated activation maps and ablation lesions under average human VT electrophysiology (EP_{avg}) and variable APD/CV conditions for model 4. **(A)** Geometric model of the infarcted heart of patient model 4. **(B)** Ablation lesions for simulations for the 5 different parameter sets. **(C)** Highlighting one VT morphologies in which the same pacing sequence applied in the same model led to the initiation of VT driven by an RD in the same ventricular region, regardless of the variability in APD/CV. t_a: activation time. The black areas in panel C for all figures are core scar – there is no electrical activation there.

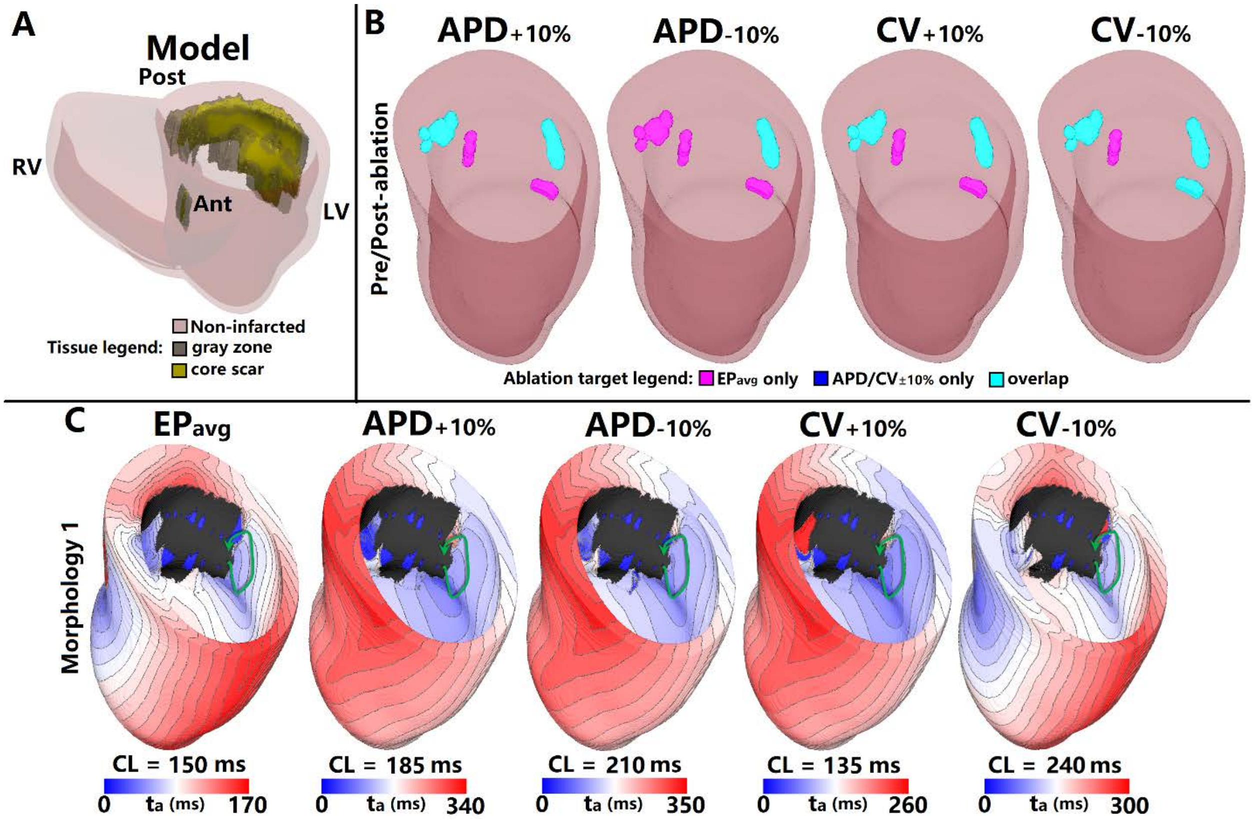

Supplement: Supplementary file 1 [file Data_Sheet_1.PDF]
